# The transcriptional STAT3 is a potential target, whereas transcriptional STAT5A/5B/6 are new biomarkers for prognosis in human breast carcinoma

**DOI:** 10.18632/oncotarget.16748

**Published:** 2017-03-31

**Authors:** Hua-Tao Wu, Jing Liu, Guan-Wu Li, Jia-Xin Shen, Yi-Teng Huang

**Affiliations:** ^1^ Department of General Surgery, The First Affiliated Hospital of Shantou University Medical College, Shantou, PR China; ^2^ Chang Jiang Scholar's Laboratory, Guangdong Provincial Key Laboratory for Diagnosis and Treatment of Breast Cancer, Shantou University Medical College, Shantou, PR China; ^3^ Open Laboratory for Tumor Molecular Biology, Department of Biochemistry, The Key Lab of Molecular Biology for High Cancer Incidence Coastal Chaoshan Area, Shantou University Medical College, Shantou, PR China; ^4^ Department of Hematology, The First Affiliated Hospital of Shantou University Medical College, Shantou, PR China; ^5^ Health Care Center, The First Affiliated Hospital of Shantou University Medical College, Shantou, PR China

**Keywords:** STAT, breast carcinoma, prognosis, Kaplan-Meier plot, transcription

## Abstract

Signal Transducer and Activators of Transcription (STAT) is a set of transcription factors, involved in diverse cellular functions. Evidences from cell lines, mouse models and human tissues implicate these transcription factors in the oncogenesis of breast cancer. However, the diverse expression patterns and prognostic values of 7 STATs remain to be elucidated. In the current study, we mined the transcriptional and survival data of STATs in patients with breast carcinoma (BC) through *ONCOMINE*, bc-GenExMiner, Kaplan-Meier Plotter and cBioPortal. It was found that STAT1/2 were up-regulated, whereas STAT3/4/5A/5B were down-regulated in BC patients compared with the normal tissues. The expressions of STAT5A/5B/6 were correlated with decreased levels of histological differentiation. In survival analyses through the *Kaplan-Meier* plotter database, high transcription levels of STAT2/4/5A/5B/6 were associated with better relapse-free survival (RFS) in all BC patients. Conversely, high STAT3 predicted shorter RFS in BC patients, suggesting that STAT3 is potential targets for precision therapy to BC patients. These data also provided STAT5A/5B/6 as new biomarker for BC prognosis.

## INTRODUCTION

Signal Transducer and Activators of Transcription (STAT) is a set of latent cytoplasmic transcription factors that are downstream factors of cytokines and enable the cell to specifically respond to changing environment. Since their identification [1], the STAT family have been recognized as critical integrators of cytokines, hormones and growth factor receptor signaling to regulate cell growth, differentiation, transformation, apoptosis, and survival. The mammary gland is the unique organ that undergoes large-scale proliferation and invasion of the fat-pad during puberty, and the STAT factors are reported to be involved in every stage of mammary gland development, as well as breast tumorigenesis [2].

Breast carcinoma (BC), one of the leading cause of cancer-related deaths among women in United States, is supposed to be a multifaceted disease of distinct biological subtypes with diverse clinical, pathological and molecular features [3]. The molecular classification of breast cancer, based on the expression of estrogen receptor/progesterone receptor (ER/PR) and epidermal growth factor receptor 2 (HER2), provided different prognostic/predictive implications and therapeutic information [4]. The steroid hormone receptors are the upstream of STAT signaling pathway and regulate STAT-dependent transcription, interestingly, STATs are capable to modulate steroid hormone-mediated transcription [5].

So far, seven STAT proteins have been identified in mammalian cells, numbered according to the order of discovery (STAT1, STAT2, STAT3, STAT4, STAT5A, STAT5B and STAT6) [2]. Among them, STAT3 and STAT5 is indicated as the oncogene in the process of pathogenesis of BC, based on evidences from breast cancer cell lines, animal models, and primary human tissues [6]. Marrero *et al*. reported that in response to angiotensin II, STAT1/2 can be rapidly tyrosine-phosphorylated, which is an important process in JAK/STAT pathway [7]. The post-translational modification may change the different conformations of STAT. For example, depending on the signal transduction pathway responsible, different effects of Prl and Src on STAT5B tyrosine phosphorylation induced the conformational differences of STAT5B, resulting selective biological responses [8]. It was reported that STAT5B signaling might promote the proliferation of ER-positive tumors [9]. On the other hand, Muller *et al*. screened and identified new loci required for JAK/STAT signaling pathway, providing molecular insights for human cancer [10]. However, the underlining mechanism which STAT is activated or depressed by different hormones and distinct STAT factors’ functions in BC has not been well elucidated.

The development of microarray technology revolutionized RNA and DNA research which has become an essential component of biology and biomedical research [11]. Based on the thousands of gene expression or copy number variation analysis published online, in this study, we performed a deep analysis of the expression and mutation of different STAT factors in BC patients, to determine their expression patterns, the potential functions and distinct prognostic values in BC.

## RESULTS

### The transcription levels of STATs in patients with breast cancer

**Figure 1:**
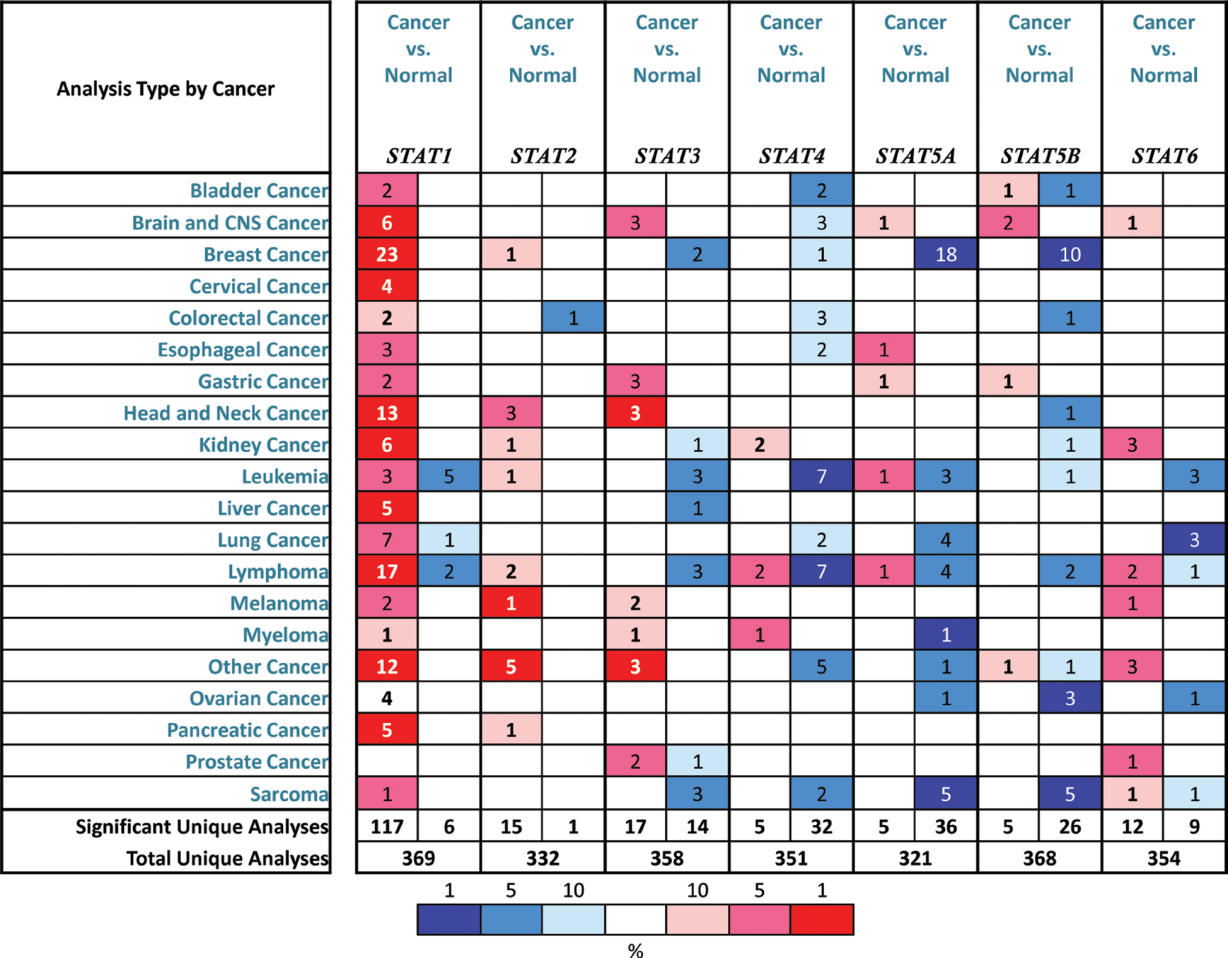
The transcription levels of STAT factors in different types of cancers This graphic was generated from *ONCOMINE*, indicating the numbers of datasets with statistically significant (*p* < 0.01) mRNA over-expression (Red) or down-expression (Blue) of STATs (different types of cancer vs. corresponding normal tissue). Cell color was determined by the best gene rank percentile for the analyses within the cell, and the gene rank was analyzed by percentile of target gene in the top of all genes measured in each research.

As mentioned before, 7 STAT factors were identified in mammalian cells. In Figure 1, we compared the transcription levels of STATs in cancers with that in normal samples, using *ONCOMINE* databases, and found that the mRNA expressions of STAT1 were significantly up-regulated in BC patients in 23 datasets. In Curtis's dataset [12], overexpression of STAT1 was found in all types of BC compared with normal samples, medullary BC with fold change = 6.511, invasive ductal BC with fold change = 3.025, invasive lobular BC with fold change = 2.223 and ductal breast carcinoma *in Situ* with fold change = 2.673 (Table 1). Another increased mRNA expression factor was STAT2 with fold change = 2.196 in patients with invasive BC, compared with normal breast tissues, reported by Cluck [13].

**Table 1:** The significant changes of STATs expression in transcription level between different types of BC and breast tissues (*ONCOMINE* database)

	Types of BC vs. Breast	Fold change	*P* value	*t*-test	Ref
STAT1					
	Medullary BC	6.511	5.38E-15	12.633	Curtis [17]
	Invasive BC	3.207	1.06E-7	7.389	
	Invasive Ductal BC	3.025	4.35E-77	28.632	
	Ductal BC	2.673	3.25E-4	4.960	
	Invasive Lobular BC	2.223	2.84E-29	12.716	
	Invasive Ductal and Invasive Lobular BC	2.247	8.41E-20	10.609	
	Total BC	2.850	3.22E-4	4.405	
STAT2					
	Invasive BC	2.196	7.74e-4	2.196	Gluck [18]
STAT3					
	Ductal BC	−2.176	1.00E-8	−7.149	Richardson [19]
	Invasive BC	−11.013	1.21E-15	−17.246	Finak [20]
STAT4					
	Mucinous BC	−2.816	0.005	−4.921	TCGA breast
STAT5A					
	Invasive Ductal BC	−2.134	7.70E-7	−7.766	Zhao [21]
STAT5B					
	Invasive Ductal BC	−2.102	3.80E-4	−6.165	Zhao

The mRNA expressions of STAT3, STAT4, STAT5A and STAT5B were found down-regulated in patients with BC. The transcription levels of STAT3 in ductal BC and invasive breast carcinoma stroma were lower than that in breast tissues (Fold changes were −2.176 and −11.013, respectively) [14, 15]. Only in TCGA breast statistics, we found that the mRNA expression of STAT4 in mucinous BC was decreased with fold change = −2.816 and *p* = 0.005. For STAT5A and STAT5B, the similar trend was found in Curtis and Zhao's datasets [12, 16]. Both STAT5A and STAT5B were significantly down-regulated in invasive ductal BC, with fold change = −2.134 and −2.102 respectively (Table 1). However, no significant difference was found between the transcription levels of STAT6 in breast cancer tissues and normal samples.

### The relationship between mRNA levels of STATs and clinicopathological parameters of BC patients

In bc-GenExMiner, the Welch's test was performed to compare the mRNA expression of STAT factors between groups of patients, according to different clinicopathological parameters. For age criterion (Table 2), there was no significant difference between ≤ 51 y and > 51 y groups, except for STAT4 with down-expression in older group. BC patients with positive nodal status showed higher STAT2 mRNA and lower STAT3/5A mRNA than negative-nodal patients.

**Table 2:** The relationship between mRNA expression of STATs and clinicopathological parameters of breast carcinoma

Variables		STAT1		STAT2		STAT3		STAT4		STAT5A		STAT5B		STAT6	
	No.*	mRNA	*p* value	mRNA	*p* value	mRNA	*p* value	mRNA	*p* value	mRNA	*p* value	mRNA	*p* value	mRNA	*p* value
Age															
≤ 51	1523	-	0.1698	-	0.0896	-	0.1564	-	0.0019	-	0.2543	-	0.2560	-	0.2137
> 51	2331	-		-		-		↓		-		-		-	
Nodal status															
–	2493	-	0.1769	-	0.0184	-	0.0008	-	0.7510	-	0.0043	-	0.1274	-	0.0563
+	1814	-		↑		↓		-		↓		-		-	
ER (IHC)															
–	1617	-	< 0.0001	-	0.1557	-	0.1056	-	< 0.0001	-	0.4237	-	< 0.0001	-	< 0.0001
+	4169	↓		-		-		↓		-		↑		↑	
PR (IHC)															
–	1076	-	< 0.0001	-	0.4143	-	0.0022	-	< 0.0001	-	0.0201	-	< 0.0001	-	< 0.0001
+	1545	↓		-		↑		↓		↑		↑		↑	
HER2 (IHC)															
–	1598	-	< 0.0001	-	0.0099	-	0.7463	-	0.0001	-	0.1000	-	0.3004	-	0.1407
+	215	↑		↑		-		↑		-		-		-	
Triple-negative Status															
Not	4349	-	< 0.0001	-	0.7214	-	0.1016	-	< 0.0001	-	0.2864	-	0.0215	-	< 0.0001
TNBC	419	↑		-		-		↑		-		↓		↓	

^*^ The number of patients was based on the group of STAT1 dataset. The exact number of patients in other group may be a little different.

ER and PR status were found to be negatively correlated with STAT1/4 expression and positively correlated with STAT5B/6 expression. For STAT3/5A, high mRNA levels were found in BC patients with PR (+). In BC patients with HER2 over-expression, the transcription levels of STAT1/2/4 were significantly increased compared with HER2-negative groups. Triple-negative breast cancer (TNBC) is a special type of BC, with ER (−), PR (−) and HER2 (−). We found that the STAT1/4 mRNA expression was significantly up-regulated in TNBC patients, whereas the STAT5B/6 mRNA expression was significantly down-regulated in TNBC patients (Table 2).

**Figure 2:**
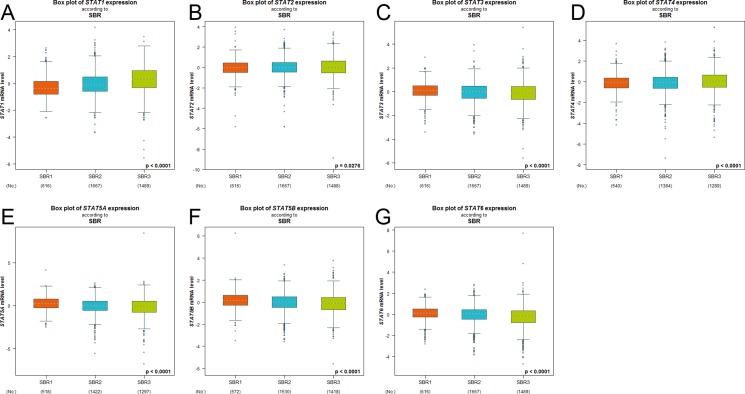
The relationship between mRNA expression of STAT factors and Scarff Bloom & Richardson grade status (SBR) Global significant different between groups was assessed by Welch's test to generate *p* value, along with Dunnett-Tukey-Kramer's tests for pairwise comparison when a global significant difference exists (*p* < 0.05).

In Scarff Bloom & Richardson grade status (SBR) criterion (Figure 2), more advanced SBR grade was associated with the higher mRNA level of STAT1 and lower mRNA levels of STAT5A/5B/6. For the STAT2/3/4, although a global significant difference was achieved, all of the groups comparison (SBR1 *vs*. SBR2, SBR1 *vs*. SBR3, and SBR2 *vs*. SBR3) of these factors did not meet the cutoff, *p* < 0.05 (Supplementary Table 1).

### Increased mRNA expressions of STAT2/4/5A/5B/6 and decreased mRNA expression of STAT3 were associated with better RFS of BC patients

**Figure 3:**
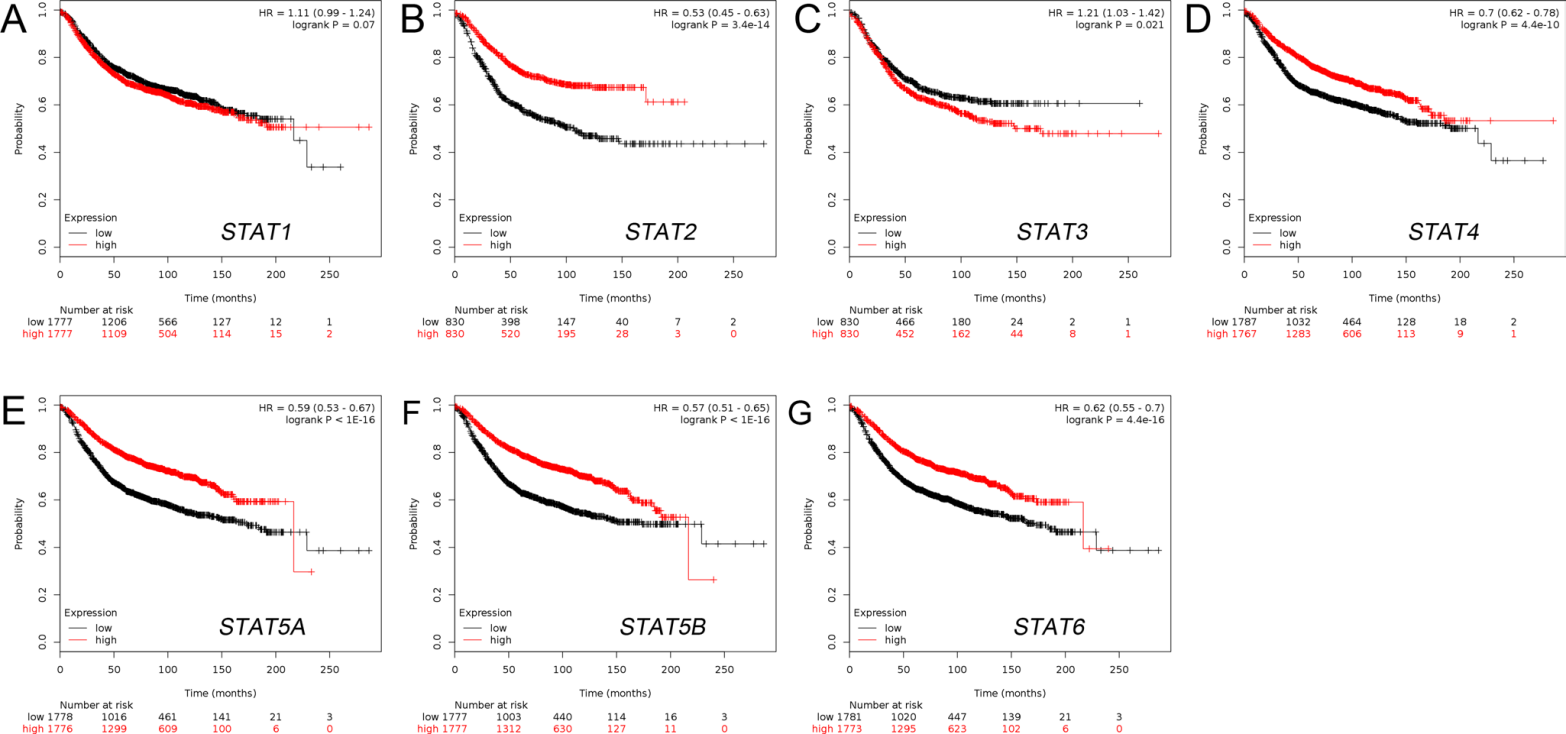
The prognostic value of mRNA level of STAT factors in BC patients (RFS in Kaplan-Meier plotter) (**A**) STAT1 (200887_s_at). (**B**) STAT2 (225636_at). (**C**) STAT3 (225289_at). (**D**) STAT4 (206118_at). (**E**) STAT5A (203010_at). (**F**) STAT5B (212549_at). (**G**) STAT6 (201331_s_at).

The Kaplan-Meier curve and log-rank test analyses revealed that STAT2/4/5A/5B/6 mRNA levels and decreased STAT3 mRNA level were significantly associated with RFS (*p* < 0.05) (Figure 3) in all BC patients. The BC patients with higher mRNA levels of STAT2/4/5A/5B/6 factors or lower mRNA level of STAT3 were predicted to have better RFS.

### The alterations in STAT factors affected the OS, not DFS in BC patients

**Figure 4:**
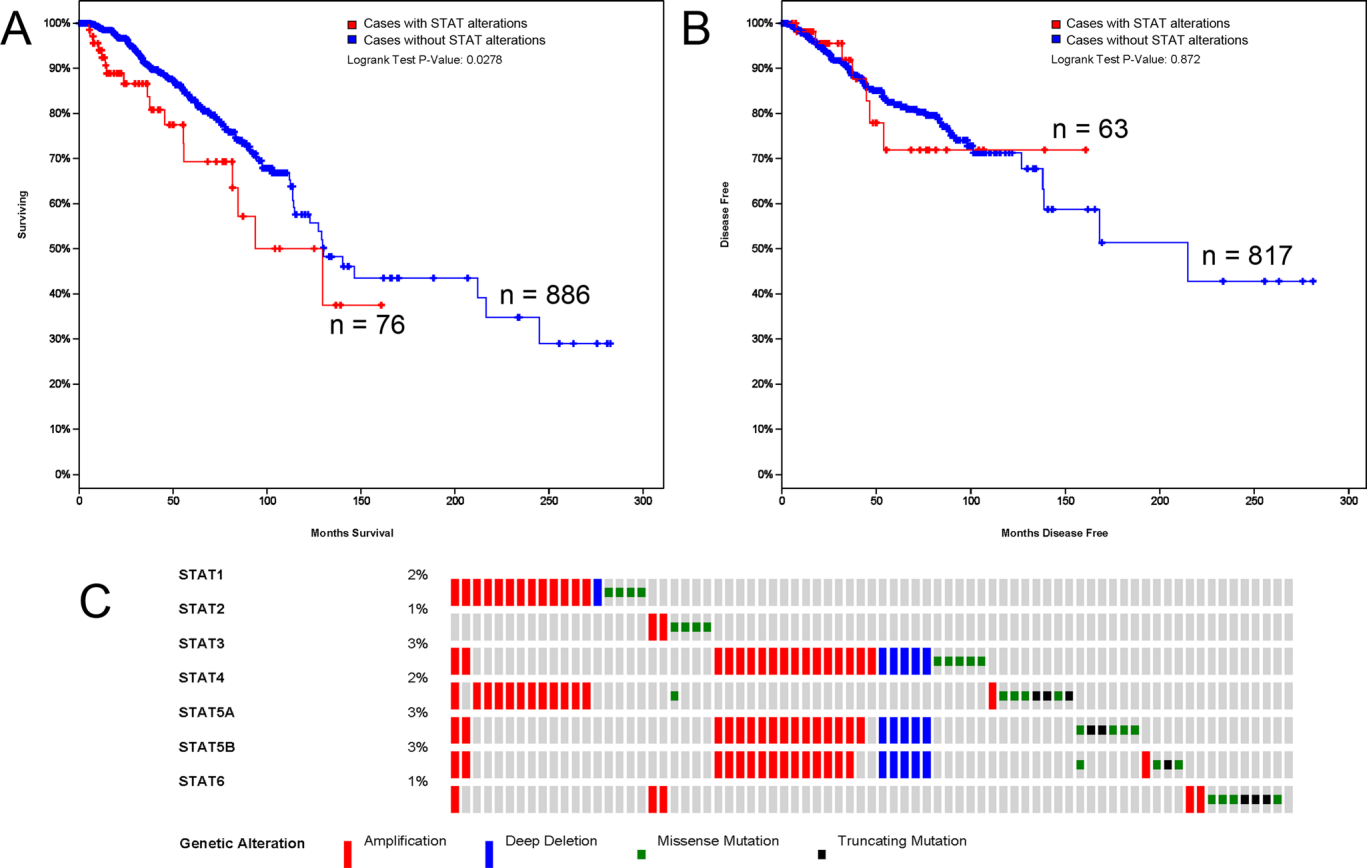
STAT genes expression and mutation analysis in breast invasive carcinoma (cBioPortal) (**A**) Kaplan-Meier plots comparing OS in cases with/without STAT alterations. (**B**) Kaplan-Meier plots comparing DFS in cases with/without STAT alterations. (**C**) Oncoprint in cBioPortal represented the proportion and distribution of samples with alterations in STAT factors. The figure was cropped on the right to exclude samples without alterations.

The alterations of STATs occurred in 76 samples out of 963 patients with breast invasive carcinoma (7%). Almost half of them (36 samples) have two or more alterations (Figure 4C). After analyzed by Kaplan-Meier plot and log-rank test, the alterations in STATs were associated with poorer overall survival (OS) in BC patients (Figure 4A). However, there was no significant difference between disease-free survival (DFS) in BC patients with/without STAT alterations (Figure 4B).

## DISCUSSION

Recently, Haricharan *et al*. provided a systematic review of STAT signaling in mammary gland differentiation, cell survival and tumorigenesis [2], indicating the importance of STAT signaling in both mammary gland and breast carcinoma development. STAT proteins, including STAT1/2/3/4/5A/5B/6, were all reported to be expressed in BC cell lines or BC tissues. However, the distinct roles of STAT factors in the development and metastasis of breast carcinoma remain to be elucidated. This study analyzed, for the first time, the mRNA expression and prognostic value of different STAT factors in breast carcinoma.

STAT1, first member of this family, is supposed to be a tumor suppressor in breast tumorigenesis. In animal model, STAT1^−/−^ may promote the spontaneous formation of breast tumors [17], with increasing susceptibility to tumorigensis initiated by ErbB2 [18] and chemical carcinogenesis [19]. However, the mRNA level of STAT1 was significantly up-regulated in breast cancer, and increased in higher SBR grade, which predicted fast-growing, spreading tumors. A DNA microarray analysis identified that RNA interference of CD24, the breast cancer stem cells (BCSC) marker, could increase the expression of STAT1, which enhanced invasiveness and superior tumorigenicity [20]. Recently, Jeon *et al*. pointed that EGFR and HER2 dimerization induced aberrant ACTA2 expression through a JAK2/STAT1 pathway, accelerating the invasiveness and metastasis of breast cancer cells [21]. Magkou *et al*. demonstrated that menopausal status affected STAT1 functions as a tumor suppressor [22], indicating the crosstalk between STATs and ER signaling pathway with unclear mechanism.

STAT2, forming heteromeric complexes with STAT1, was found in MCF7 cells, an ER+ human BC cell line [23]. In the current study, STAT2 was increased in BC tissues, similar with STAT1. However, the BC patient with higher mRNA levels of STAT2 was predicted with better RFS. On the protein level, Ogony *et al*. recently reported that overexpression of IFITM1 enhances the aggressive phenotype of TNBC cells in STAT2-dependent manner [24]. As few evidences focused on STAT2, the underlining function of STAT2 needs more researches.

Constitutive STAT3 activation was reported with various human cancers, usually with poor outcome, as a promising target for cancer therapy [25]. Not surprisingly, the transcription level of STAT3 was negatively associated with RFS of BC patients, consistent with the protein level. STAT3 transactivates proliferative genes (*cMyc* and *Cyclin D1*), prosurvival genes (*Bcl-xl* and *Survivin*) and invasive genes (*VEG-f* and *Klf-8*) [26], resulting in rapidly growing tumors with highly metastastic capacity. In transgenic mice model, STAT3 also establishes an inmuunosuppressive microenvironment during the early stages of breast carcinogenesis to promote tumor develop and metastasis [27]. Zhang *et al*. recently found that STAT3 could bind promoter region of TXNDC17 and up-regulated its expression, mediating Taxol resistance via enhancing autophagy in human colorectal cancer cells [28], which may be also involved in the mechanism of chemoresistance in BC patients, whereas Bui *et al*. found that Notch4/STAT3 signaling induced the epithelial-mesenchymal transition in tamoxifen-resistant human breast cancer, increasing micrometastatic tumor burden [29].

MCF7 cells also expressed STAT4 [23], without specific evidence of STAT4 expression in human BC. We found that STAT4 was down-regulated in BC patients and higher STAT4 expression predicted better RFS. However, considering hormone receptor criterions, STAT4 showed positive correlation with TNBC, a more aggressive type with poor prognosis. Crytotanshinone (CPT) showed potential therapeutic value in animal breast model through enhancing cytotoxic CD4+ T cells by up-regulating JAK2 and STAT4 phosphorylation [30], suggesting that STAT4 may be play opposite function with STAT3 in human cancers.

Constitutive STAT5 activation is also observed in the majority of leukemias and many solid tumors, encoding by *Stat5a* and *Stat5b* genes on human chromosome 17 in a locus that also contains the *Stat3* gene [31]. STAT5A and STAT5B are closely related members with 96% conserved at the protein level, and the highest degree of divergence is found in the C-terminal transactivation domain. Not surprisingly, both of their expressions were found to be suppressed in BC patients, especially with higher SBR grade. Importantly, the BC patient with higher STAT5A or STAT5B expression showed better RFS, providing a potential prognostic biomarker for BC. Yamashita *et al*. demonstrated that STAT5 expression is a predictive factor for endocrine therapy response and a strong prognostic molecular marker in ER+ breast cancer, predicting BC patients who may benefit from endocrine therapy [32]. The hormone prolactin (PRL) recruited STAT5 to increase transcription and cell proliferation, through dissociating Histone H1 mediated by chromosomal protein HMGN2 [33], providing a novel mechanism of full STAT5 recruitment.

At present, the research of STAT6 is primarily described in human breast cell lines and expressed in MCF7, like STAT2/4 [34]. Although no significant difference was found between the STAT6 expression in BC and normal tissues, the BC patients with high STAT6 expression level was predicted to have better survival in this study. The expression of STAT was correlated with increased levels of histological differentiation (SBR). STAT6 is induced by and required for IL-4-mediated growth inhibition and induction of apoptosis in human breast cancer cells [34, 35], confirming its anti-tumor function in BC. Recently, STAT6-TP63 pathway was reported to be activated by IL12Ralpha2, suppressing breast cancer lung metastasis [36]. STAT6 was found to be a novel target of PVT1-derived miR-1207-5p, and miR-1207-5p promoted breast cancer cell growth by targeting STAT6, which in turn control CDKN1A and CDKN1B [37], confirming the tumor suppressing role of STAT6 in breast cancer.

Although the alterations occurred in STATs affected the OS in BC patients, the main contributor and underling mechanism is still unclear. Vaclavicek *et al*. investigated the polymorphisms in JAK2 and STAT3/5A/5B genes in a case-control study and manifested putative association of STAT polymorphisms with increased risk odds ratio [38]. Recently, Slattery *et al*. examined 12 genes in JAK/STAT/SOCS signaling pathway with breast cancer risk and mortality and found that STAT6 rs3024979 and TYK2 rs280519 altered breast cancer-specific mortality among all women [39]. So the exact mechanism of STAT alteration with shorter OS in BC patients needs more investigations.

In this study, we systemically analyzed the expression and prognostic value of STATs in breast carcinoma, providing better understanding of the heterogeneity and complexity in the molecular biology of BC. Our finding supports that STAT3 was the potential treated target for breast cancer therapy, whereas STAT5A/5B/6 were potential prognostic markers for better survival of BC, providing more accurate prognosis.

## MATERIALS AND METHODS

### Ethics statement

This study was approved by the Academic Committee of Shantou University Medical College, and conducted according to the principles expressed in the Declaration of Helsinki. All the datasets were retrieved from the publishing literature, so it was confirmed that all written informed consent was obtained.

### ONCOMINE analysis

*ONCOMINE* gene expression array datasets (www.oncomine.org), an online cancer microarray database [40], was used to analyze the transcription levels of STATs in different cancers. The mRNA expressions of STATs in clinical cancer specimens were compared with that in normal controls, using a Students’ *t*-test to generate a *p* value. The cut-off of *p* value and fold change were defined as 0.01 and 2, respectively.

### Breast cancer gene-expression miner v4.0

Breast Cancer Gene-Expression Miner v4.0 (bc-GenExMiner v4.0) consisted 36 annotated genomic datasets and three statistical mining functions [41, 42]. The expression module was added on 2016/03/29, comparing the expression of a target gene according to clinical criteria, such as hormonal receptors, nodal status, and so on. The prognostic module assessed the prognostic impact of candidate genes in human BC and provided potential prognostic markers for BC. The correlation module computed the correlation between genes or identified clusters of correlated co-expressed genes located in the same chromosomal region.

### The kaplan-meier plotter

The prognostic value of STATs mRNA expression was evaluated using an online database, Kaplan-Meier Plotter (www.kmplot.com) [43], which contained gene expression data and survival information of 4,142 clinical breast cancer patients [44]. To analyze the relapse free survival (RFS) of patients with BC, patient samples were split into two groups by median expression (high vs. low expression) and assessed by a Kaplan-Meier survival plot, with the hazard ratio (HR) with 95% confidence intervals (CI) and logrank *p* value. Only the JetSet best probe set of STATs were chosen to obtain Kaplan-Meier plots in which the Number-at-risk is indicated below the main plot.

### TCGA data and cBioPortal

The Cancer Genome Atlas had both sequencing and pathological data on 30 different cancers [45]. The breast invasive carcinoma (TCGA, Provisional) dataset including data from 1105 cases with pathology reports was selected for further analyses of STATs using cBioPortal (www.cbioportal.org) [46, 47]. The genomic profiles included mutations, copy-number variance (CNV) from GISTIC, mRNA expression z-scores (RNA Seq V2 RSEM) and protein expression z-scores (RPPA). OS and DFS were calculated according to the cBioPortal's online instruction.

## SUPPLEMENTARY MATERIALS TABLES



## Abbreviations

BC: Breast Carcinoma
BCSC: Breast Cancer Stem Cells
CI: Confidence Intervals
CNV: Copy-Number Variance
CPT: Crytotanshinone
DFS: Disease-Free Survival
ER: Estrogen Receptor
HER2: Epidermal Growth Factor Receptor 2
HR: Hazard Ratio
OS: Overall Survival
PR: Progesterone Receptor
RFS: Relapse-Free Survival
SBR: Scarff Bloom & Richardson grade
STAT: Signal Transducer and Activators of Transcription
TCGA: The Cancer Genome Atlas
TNBC: Triple-Negative Breast Cancer
