# Supplementary material for: The transcriptional STAT3 is a potential target, whereas transcriptional STAT5A/5B/6 are new biomarkers for prognosis in human breast carcinoma
Source: Oncotarget. 2017 Mar 31;8(22):36279–88. doi: 10.18632/oncotarget.16748 (PMC5482654; doi:10.18632/oncotarget.16748)
Supplement: Supplementary file 1 [file oncotarget-08-36279-s001.pdf]

The transcriptional STAT3 is a potential target, whereas transcriptional STAT5A/5B/6 are new biomarkers for prognosis in human breast carcinoma

Supplementary Materials

Supplementary Table 1: The results of Dunnett-Tukey-Kramer's test for pairwise comparison in SBR criterion

mRNA	Pairwise comparison of SBR			*P* value
STAT1	SBR2	>	SBR1	< 0.0001
	SBR3	>	SBR1	< 0.0001
	SBR3	>	SBR2	< 0.0001
STAT2	SBR2	=	SBR1	> 0.10
	SBR3	>	SBR1	< 0.05
	SBR3	=	SBR2	> 0.10
STAT3	SBR2	<	SBR1	< 0.0001
	SBR3	<	SBR1	< 0.0001
	SBR3	=	SBR2	> 0.10
STAT4	SBR2	=	SBR1	> 0.10
	SBR3	>	SBR1	< 0.001
	SBR3	>	SBR2	< 0.001
STAT5A	SBR2	<	SBR1	< 0.0001
	SBR3	<	SBR1	< 0.0001
	SBR3	<	SBR2	< 0.05
STAT5B	SBR2	<	SBR1	< 0.0001
	SBR3	<	SBR1	< 0.0001
	SBR3	<	SBR2	< 0.001
STAT6	SBR2	<	SBR1	< 0.01
	SBR3	<	SBR1	< 0.0001
	SBR3	<	SBR2	< 0.0001
